# Supplementary material for: An Isocaloric Nordic Diet Modulates RELA and TNFRSF1A Gene Expression in Peripheral Blood Mononuclear Cells in Individuals with Metabolic Syndrome—A SYSDIET Sub-Study
Source: Nutrients. 2019 Dec 3;11(12):2932. doi: 10.3390/nu11122932 (PMC6950764; doi:10.3390/nu11122932)
Supplement: Supplementary file 1 [file nutrients-11-02932-s001.pdf]

Supplemental Table S1: Gene expression change from baseline to end of study in SYSDIET relative to control group.

Gene	Estimate	95% CI	P-value	FDR
ABCA1	0.321707	(−0.06 - 0.71)	0.101	0.590
ABCG1	−0.0868	(−0.49 - 0.32)	0.671	0.801
CCL2	0.119038	(−0.41 - 0.65)	0.655	0.801
CCL5	0.079275	(−0.28 - 0.44)	0.660	0.801
CCR2	0.324836	(−0.06 - 0.71)	0.096	0.590
CCR4	0.072374	(−0.32 - 0.46)	0.714	0.801
CD36	−0.22348	(−0.58 - 0.13)	0.210	0.590
CD40	−0.30838	(−0.7 - 0.08)	0.122	0.590
CD40LG	−0.24171	(−0.57 - 0.09)	0.147	0.590
CPT1A	−0.15937	(−0.56 - 0.24)	0.427	0.705
CPT1B	−0.18168	(−0.7 - 0.34)	0.490	0.735
CRAT	−0.06342	(−0.42 - 0.29)	0.721	0.801
CXCR2	0.370024	(−0.03 - 0.77)	0.067	0.590
HMGCR	−0.20361	(−0.53 - 0.12)	0.218	0.590
ICAM1	−0.09487	(−0.43 - 0.24)	0.578	0.782
IFNG	−0.23788	(−0.65 - 0.17)	0.252	0.590
IKBKB	0.272156	(−0.12 - 0.66)	0.166	0.590
IL18	−0.40786	(−1.03 - 0.22)	0.199	0.590
IL1B	0.210775	(−0.28 - 0.7)	0.391	0.705
IL1RN	−0.13093	(−0.47 - 0.2)	0.438	0.705
IL23A	−0.05431	(−0.38 - 0.28)	0.744	0.801
IL23R	0.07138	(−0.35 - 0.49)	0.735	0.801
IL6	0.136603	(−0.29 - 0.57)	0.530	0.742
IL8	0.64915	(−0.15 - 1.45)	0.110	0.590
LDLR	−0.02874	(−0.36 - 0.3)	0.863	0.884
MMP9	0.309196	(−0.29 - 0.91)	0.308	0.616
NAMPT	−0.22543	(−0.63 - 0.18)	0.275	0.590
NFKBIA	−0.17952	(−0.49 - 0.13)	0.249	0.590
PDGFA	−0.26867	(−0.68 - 0.15)	0.200	0.590
PDGFB	−0.29906	(−0.64 - 0.04)	0.087	0.590
PDK4	−0.14372	(−0.48 - 0.19)	0.397	0.705
PLIN2	−0.17631	(−0.5 - 0.15)	0.281	0.590
PPARA	0.108198	(−0.22 - 0.44)	0.515	0.742
PPARD	−0.11639	(−0.42 - 0.19)	0.453	0.705
RELA	0.414439	(0.08 - 0.75)	0.016	0.345
SREBF1	0.221734	(−0.35 - 0.79)	0.442	0.705
TGFB2	0.048171	(−0.42 - 0.52)	0.840	0.882
TLR4	0.007217	(−0.33 - 0.35)	0.966	0.966
TNF	−0.20531	(−0.58 - 0.16)	0.273	0.590
TNFRSF1A	−0.51981	(−0.87 - −0.17)	0.004	0.186
TNFRSF1B	0.059178	(−0.28 - 0.4)	0.727	0.801
UCP2	−0.27445	(−0.65 - 0.1)	0.146	0.590

Difference between SYSDIET and control group analysed with a linear regression model, adjusted for age, sex and study center.

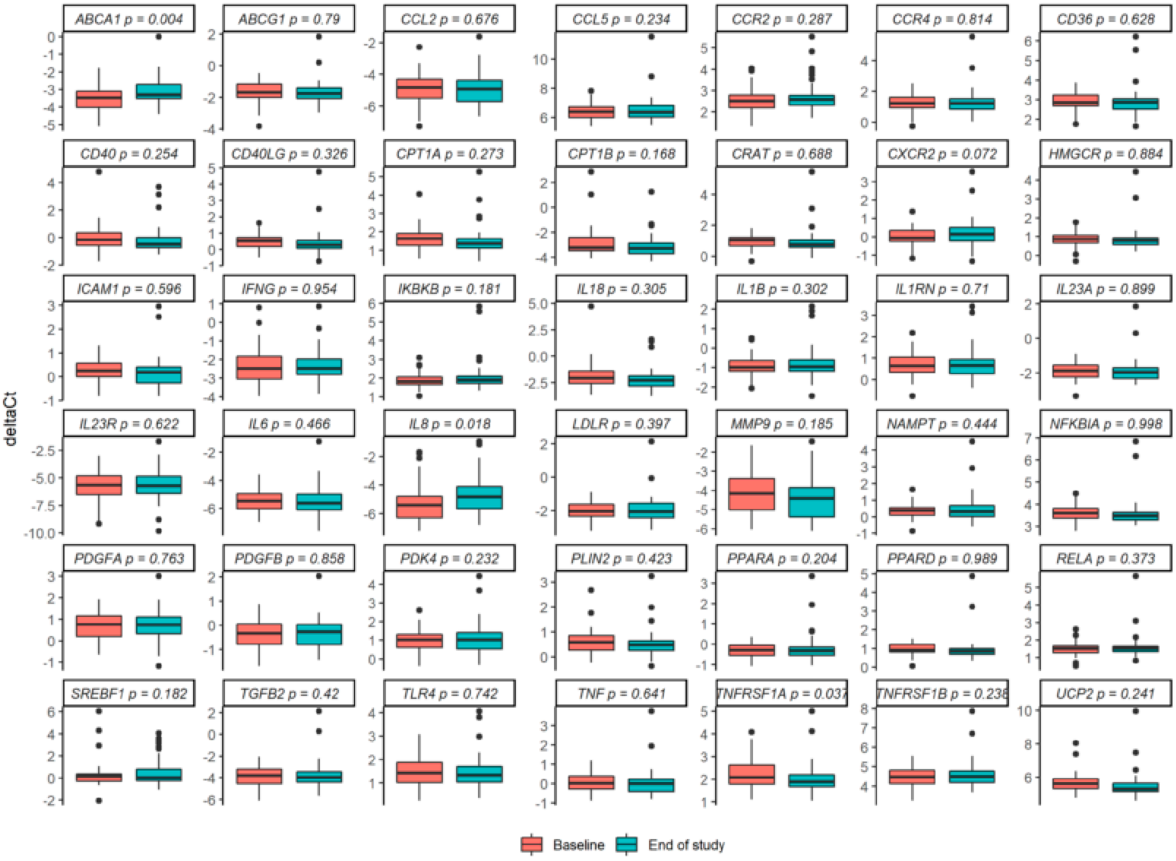

Figure S1. Gene expression changes (deltaCt) in the ND group at baseline and end of study. ΔCt was calculated as $Ct_{(reference\ gene)}$–$Ct_{(target)}$, and the log ratio (ΔΔCt) was calculated as $ΔCt_{(end\ of\ study)}$–$ΔCt_{(baseline)}$. Differences between the time points are tested with a paired *t*-test. *p*-values < 0.05 were considered significant.

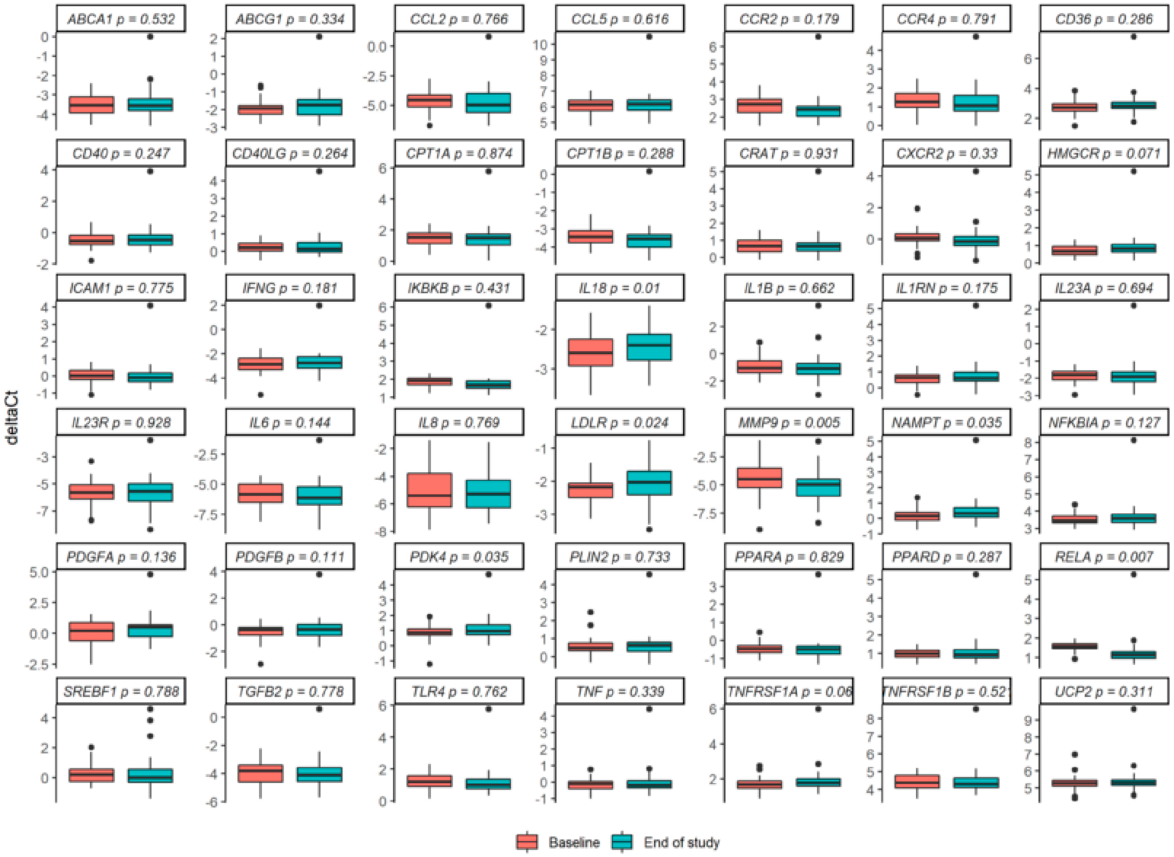

Figure S2. Gene expression changes (deltaCt) in the CD group at baseline and end of study. ΔCt was calculated as $Ct_{(reference\ gene)}$–$Ct_{(target)}$, and the log ratio (ΔΔCt) was calculated as $\Delta Ct_{(end\ of\ study)}$–$\Delta Ct_{(baseline)}$. Differences between the time points are tested with a paired *t*-test. *p*-Values < 0.05 were considered significant.
